# Mtmr8 is essential for vasculature development in zebrafish embryos

**DOI:** 10.1186/1471-213X-10-96

**Published:** 2010-09-05

**Authors:** Jie Mei, Sha Liu, Zhi Li, Jian-Fang Gui

**Affiliations:** 1 State Key Laboratory of Freshwater Ecology and Biotechnology, Center for Developmental Biology, Institute of Hydrobiology, Chinese Academy of Sciences, Graduate School of the Chinese Academy of Sciences, Wuhan 430072, China

## Abstract

**Background:**

Embryonic morphogenesis of vascular and muscular systems is tightly coordinated, and a functional cooperation of Mtmr8 with PI3K in actin filament modeling and muscle development has been revealed in zebrafish. Here, we attempt to explore the function of Mtmr8 in vasculature development parallel to its function in muscle development.

**Results:**

During early stage of somitogenesis, *mtmr8 *expression was detected in both somitic mesodem and ventral mesoderm. Knockdown of *mtmr8 *by morpholino impairs arterial endothelial marker expression, and results in endothelial cell reduction and vasculogenesis defects, such as retardation in intersegmental vessel development and interruption of trunk dorsal aorta. Moreover, *mtmr8 *morphants show loss of arterial endothelial cell identity in dorsal aorta, which is effectively rescued by low concentration of PI3K inhibitor, and by over-expression of *dnPKA *mRNA or *vegf *mRNA. Interestingly, *mtmr8 *expression is up-regulated when zebrafish embryos are treated with specific inhibitor of Hedgehog pathway that abolishes arterial marker expression.

**Conclusion:**

These data indicate that Mtmr8 is essential for vasculature development in zebrafish embryos, and may play a role in arterial specification through repressing PI3K activity. It is suggested that Mtmr8 should represent a novel element of the Hedgehog/PI3K/VEGF signaling cascade that controls arterial specification.

## Background

MTM (myotubularin myopathy) family factors are members of the growing class of dual-specificity phosphatases (DSPs) including PTEN, which can dephosphorylate the products of phosphoinositide 3-kinases (PI3K), and are negative regulators of the PI3K/Akt signaling pathways[1]. A potential function for PI3K and PTEN has been suggested in both angiogenic signaling[2,3] and various models of muscle defects[4]. And, dual-specific phosphatase-5 (Dusp-5) has been identified to play a functional role in vascular development through counteracting the function of Snrk-1, a serine threonine kinase in angioblast development[5]. However, functions of MTM family members in these processes are not clear.

In vertebrates, vascular and muscular systems are tightly connected. And, the cells in dorsal aorta and myotomal muscle are both derived from mesoderm. Endothelial cells (ECs) forming intersegmental vessel (SE) migrate following a path initially along the somite boundary and later between notochord and somitic tissue[6]. These data suggested that a dynamic connection between somitogenesis and vasculature development might exist, and the connection might be influenced by some signaling molecules. For example, semaphorin-plexin-signaling was proved to play significant roles in both somitogenesis and vasculature formation in zebrafish[6]. Furthermore, zebrafish *perlecan *was demonstrated to play a central function in skeletal muscle and cardiovascular development[7].

Several signaling pathways, such as vascular endothelial growth factor (VEGF) signaling, and PI3K/Akt and Hedgehog (Hh) signaling which genetically interacts with each other[8-10], had been shown to be involved in vascular formation. *In vitro *studies suggested a role for AKT/PKB as a downstream effector of VEGF signaling[11,12]. Moreover, over-expression of the downstream effector AKT/PKB rescued VEGF receptor blockade[13]. In addition, activation of protein kinase A (PKA), a negative regulator of hedgehog pathway, effectively inhibited Akt, which demonstrated a direct role for AKT in regulating Hedgehog signaling[13,14]. Myocardial Hh activation triggered by FGF signaling, is essential for Vegf expression[15]. However, these signaling interactions form complicated network and need further explanation.

Specific genes controlling artery/vein specification have been identified in different vertebrate species[16]. In zebrafish embryo, sonic hedgehog (Shh) and Vegf, expressed in the notochord and somite respectively, are required for arterial specification[9,17]. Conversely, activation of PI3K/AKT signaling in angioblasts promotes venous specification[18]. However, the precise mechanism of signaling interactions in vascular development remains to be elucidated. Interestingly, our recent study has shown a functional cooperation of the MTM family member Mtmr8 with PI3K in muscle development in zebrafish, and revealed a possible participation of Mtmr8 in the Hedgehog (Hh) pathway[19]. To explore the function of Mtmr8 in vasculature development, here, we further observed the vascular development defects of Mtmr8 loss-of-function. Subsequently, we found that the Mtmr8, as a negative regulator of PI3K, affected the Hedgehog and Vegf pathway in the blood vessel development *in vivo*. The data indicate that Mtmr8 is essential for vasculature development in zebrafish.

## Results

### Developmental defects of hemato-vascular progenitors in mtmr8 morphants

**Figure 1:**
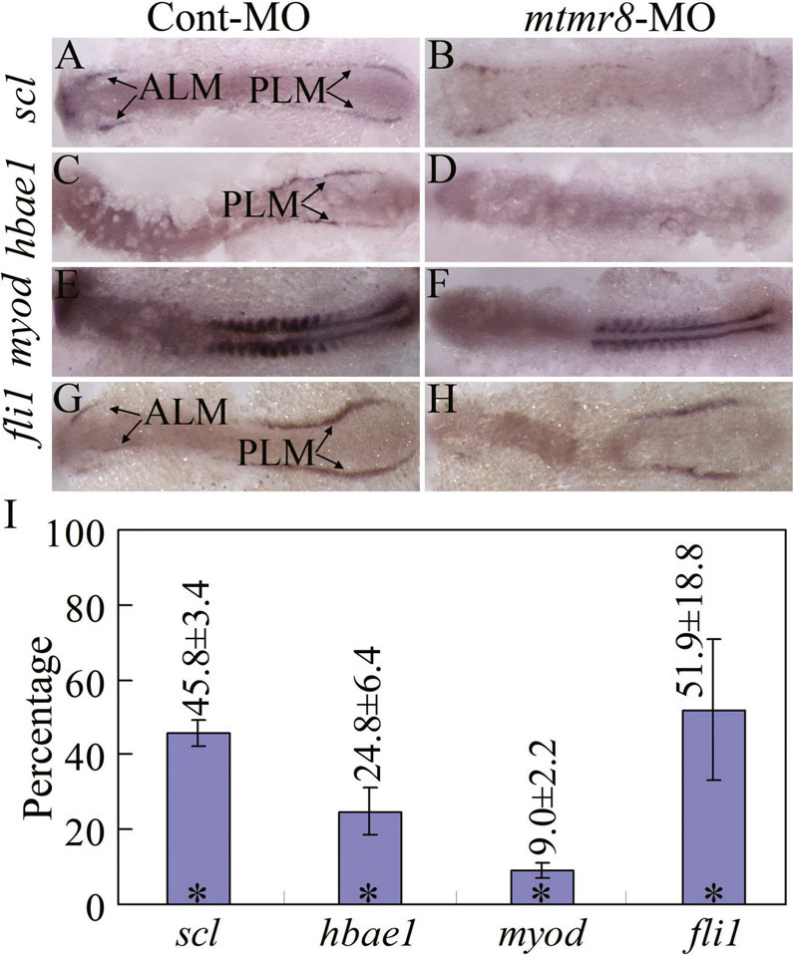
**Early marker genes for hemato-vascular progenitors, such as early angioblast/hematopoietic mesoderm marker genes *scl *(A, B) and *hbae1*(C, D), somitic mesoderm marker gene *myod*(E, F), and endothelial cell marker gene *fli1*(G, H), are affected in lateral mesoderm of *mtmr8 *knockdown embryos during somitogenesis**. All pictures were taken from 12 somites stage embryos. Panels are flat-mounted dorsal views (anterior toward the left). ALM (anterior lateral mesoderm) and PLM (posterior lateral mesoderm) are indicated by arrows. (I) Percentages of *scl*, *hbae1*, *myod *and *fli1 *expression of *mtmr8 *morphant embryos relative to Cont-MO embryos revealed by Q-RT-PCR. * indicates significance of *p *< 0.05.

During zebrafish somitogenesis, *mtmr8 *transcript was detected in early somitic mesoderm between 1-13 somites, and later in ventral mesoderm[19], where multipotential progenitors gives rise to at least two different tissues: the hematopoietic and endothelial lineages[20]. In order to reveal further functions of Mtmr8, we analyzed the expression pattern of some known early marker genes for hemato-vascular progenitors in lateral mesoderm of *mtmr8 *morphants by *in situ *hybridization. As shown in Figure 1, the early angioblast/hematopoietic mesoderm marker genes, *scl *and *hbae1*, are expressed in anterior lateral mesoderm (ALM) and/or posterior lateral mesoderm (PLM) in Cont-MO injected embryos (Figure 1A, C), whereas at the same embryonic stage of 12 somites, significant expression reduction is observed in *mtmr8 *morphants (Figure 1B, D). And, Mtmr8 knockdown simultaneously affects the formation of somitic mesoderm, because *myod *expression is significantly reduced in *mtmr8 *morphants in comparison to Cont-MO embryos (Figure 1E, F). As *Scl *was demonstrated to be very critical for zebrafish endothelial and artery development[21], we further assayed expression of *fli1*, a marker for endothelial cells and also for a subset of the hematopoietic cells in early development stage[22]. Compared to Cont-MO embryos (Figure 1G), *fli1 *is also significantly reduced in anterior lateral mesoderm (ALM) of the *mtmr8 *morphants, whereas its expression defect is mild in posterior lateral mesoderm (PLM) (Figure 1H). Moreover, the expression differences of these marker genes were confirmed by quantitative RT-PCR. As shown in Figure 1I, the expression levels of *scl*, *hbae1*, *myod*, and *fli1 *are greatly reduced to 45.8%, 24.8%, 9.0%, and 51.9% of the control embryos. The data indicate that Mtmr8 knockdown affects endothelial cell formation and results in developmental defects of hemato-vascular progenitors.

### Determination of abnormal vascular phenotypes in mtmr8 morphant embryos

**Figure 2:**
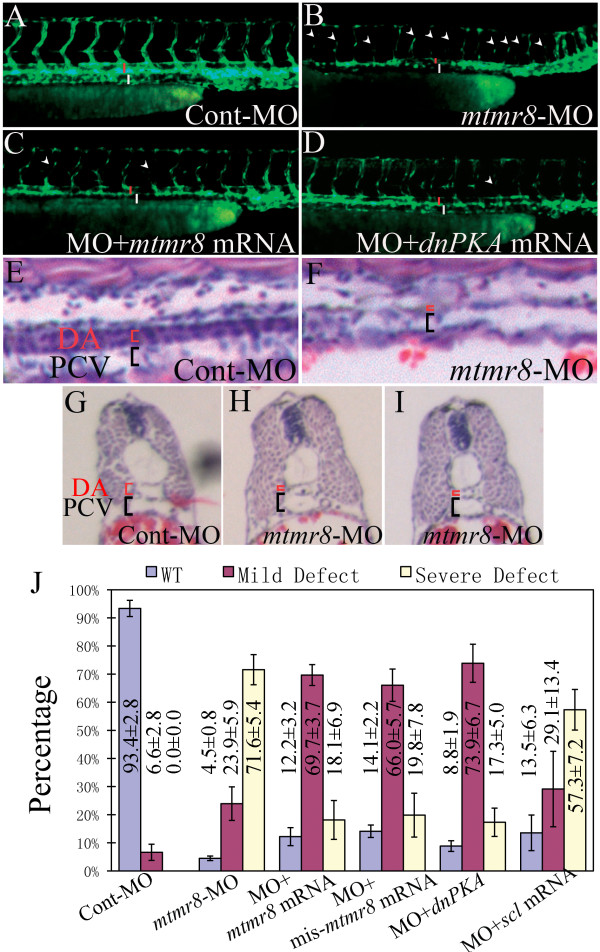
**Mtmr8 knockdown results in vascular defects**. (A-D) Vascular morphology in Cont-MO (A), *mtmr8*-MO (B), *mtmr8*-MO+ *mtmr8 *mRNA (C) and *mtmr8*-MO+*dnPKA *mRNA *Tg(kdrl:GFP)^la116 ^*embryos at 48 hpf. The presumptive lumen of DA and PCV are indicated by red and white bars, respectively. White arrowheads point to the interruption of Se. (E, F) Longitudinal sections of trunk regions in 48 hpf corresponding embryos. (G-I) Transverse sections of trunk regions in 48 hpf corresponding embryos. Sections were stained with hematoxylin and eosin. (J) Statistical data of three different defects in three independent experiments of Cont-MO, *mtmr8*-MO, *mtmr8*-MO+*mtmr8 *mRNA, *mtmr8*-MO+mis-*mtmr8 *mRNA, *mtmr8*-MO+*dnPKA *mRNA, and *mtmr8*-MO+*scl *mRNA.

Previous study has observed that *mtmr8 *is expressed predominantly in the vasculature around and after 24 hpf[19]. To determine whether Mtmr8 is required for vasculogenesis and angiogenesis, transgenic *Tg(kdrl:GFP)^la116^*[23] embryos were injected with morpholino against *mtmr8 *as described previously[19], and their vasculature development was assayed. As shown in Figure 2, in comparison with normal vasculature phenotypes in Cont-MO embryos (Figure 2A), the primary intersegmental vessel (Se)[24] and dorsal longitudinal anastomotic vessel (DLAV) can sprout and form, but intensive defects are observed in *mtmr8-*MO morphants at 48 hpf. The vascular plexus in tail seems to be less complex and becomes narrow. Significantly, the *mtmr8 *morphants display a remarkable reduction in the size of dorsal aorta (DA), and the DA size reduction degree is correlated with severity of the impaired Se (Figure 2B). The defected embryos are divided into three kinds according to relative normal Se number. The severely defected embryos with less than 5 normal Se and mild defected embryos with more than 5 normal Se in the trunk are 71.6% and 23.9% respectively, whereas normal phenotype is only 4.5% (Figure 2J). To demonstrate the defected specificity, we co-injected 100 pg of zebrafish *mtmr8 *mRNA with the MO, and thereby led to an increase percentage of embryos in mild (69.7%) and normal (18.1%) phenotypes (Figure 2C, J). And, we co-injected a mis-match *mtmr8 *mRNA which is unable to bind MO because of the existence of nucleotide substitutions but still encodes the same protein, with the MO, and could also rescue the morphant (Figure 2J). Previous study observed that the expression of *ptc1*, a downstream target gene of the Hedgehog signaling pathway, was reduced in *mtmr8 *morphants, which could be rescued by co-injection with *dnPKA *mRNA[19]. And, the Hedgehog signaling pathway was shown to be essential for vasculture differentiation and to induce the expression of Vegf during the same process[9]. Consistently, the vascular defect of *mtmr8 *morphants could be rescued by co-injection of 100 pg *dnPKA *mRNA (Figure 2D, J). And, *scl *overexpression could partially rescue the vascular defect of *mtmr8 *morphants, even though the defect reduction was very small (Figure 2J). Moreover, HE stained longitudinal and transverse sections were analyzed. In comparison with that of Cont-MO embryos (Figure 2E, G), the DA appears more narrow in the *mtmr8 *morphants (Figure 2F, H, I). These data indicate that Mtmr8 is essential for the integrity of muscle and vasculature development, and that hedgehog pathway mediates these biological activities.

**Figure 3:**
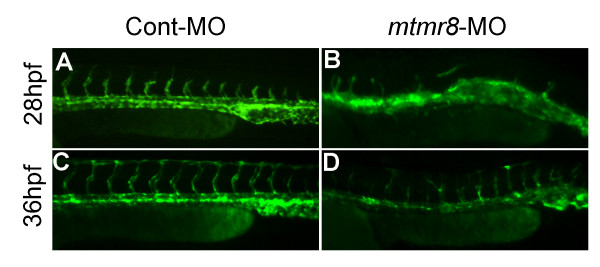
**The effects of *mtmr8 *knockdown on early vasculature development and blood formation in *Tg(kdrl:GFP)^la116 ^*transgenic embryos**. (A-D) Vascular morphology in Cont-MO (A, C) and *mtmr8*-MO (B, D) *Tg(kdrl:GFP)^la116 ^*transgenic embryos at 28 hpf (A, B) and 36 hpf (C, D), respectively.

To clarify what stage of vasculogenesis is affected, we further observed blood circulation and vascular phenotypes in early morphant embryos. The movies at same stages of 28 hpf (Additional files 1) and 36 hpf (Additional files 2) reveal significant difference of blood cell circulation between normal control embryos and morphant embryos. In Cont-MO embryos, blood cells normally circulate in the trunk vessels, whereas almost no any blood cells flow in that of 28 hpf morphant embryos (Additional files 1), and only a few of blood cells move through arteries and veins in that of 36 hpf morphant embryos (Additional files 2). We also checked the cardiac system, however, no obvious cardiac defect was observed in the *mtmr8 *morphants (Additional files 3). Moreover, abnormal vasculogenesis was early observed in *mtmr8 *morphant embryos of *Tg(kdrl:GFP)^la116 ^*transgenic zebrafish. As shown in Figure 3, when Se sprouts have produced at 28 hpf and complete vascular system has formed at 36 hpf in Cont-MO embryos (Figure 3A, C), the corresponding vascular structures are absent or abnormal at the same stages in the *mtmr8 *morphant embryos (Figure 3B, D). The above data further indicate that the vascular development defect begins from the early stage, because the down-regulation of multiple vascular marker genes for vasculogenesis has been observed in the *mtmr8 *morphant embryos.

### Expression defects of molecular marker genes for artery/vein and vascular endothelium in mtmr8 morphants

**Figure 4:**
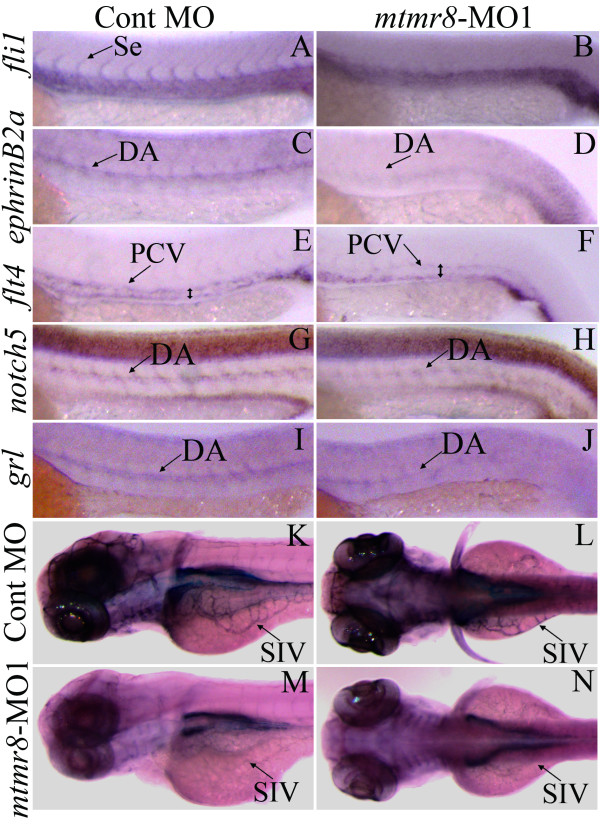
**The effects of *mtmr8 *knockdown on vascular marker genes and vascular endothelium**. (A-J) Whole mount *in situ *hybridization with the vascular marker gene-specific probes (as shown on the left) in Cont-MO (A, C, E, G and I) and *mtmr8*-MO (B, D, F, H and J) embryos at 26 hpf. The signals of DA, PCV and Se are indicated by arrows, and the PCV expansion size is indicated by double-arrowheads in the *flt4 *probe hybridization (E and F). (K-N) Lateral (K, M) and dorsal (L, N) views of alkaline phosphatase-stained Cont-MO (K, L) and *mtmr8*-MO (M, N) embryos at 3 dpf.

To further reveal molecular expression defects, we firstly used the endothelial cell marker *fli1 *to monitor the effect of *mtmr8 *knockdown on vascular development. As shown in Figure 4, in Cont-MO embryos, *fli1 *is abundantly expressed in axial vasculature and Se sprouts from the dorsal aorta at 26 hpf (Figure 4A), whereas in *mtmr8 *morphants at the same stage, little *fli1 *transcript is observed in the corresponding axial vasculature and Se sprout regions (Figure 4B). The data indicates that the endothelial-cell differentiation giving rise to the axial vasculature is defective in *mtmr8 *morphants. As the later-forming Se and new vessel growth were believed to form respectively by sprouting and from preexisting vessels[25], we next checked the formation of primary vasculature. The expression of *ephrinB2a*, an artery marker, was detected in presumptive DA region of Cont-MO embryos (Figure 4C). However, its expression was severely reduced in the *mtmr8 *morphants (Figure 4D), suggesting that the artery development should be impaired. The expression of vein marker *flt4 *was observed in PCV region of Cont-MO embryos (Figure 4E), whereas the *flt4*-positive PCV regions were expanded, and its expression was partially reduced in the *mtmr8 *morphants (Figure 4F). To confirm the role of Mtmr8 in artery formation, we further examined the expression changes of two Notch signaling markers *notch5 *and *grl/hey2*. As shown in Figure 4G-J, significant expression decrease of the two arterial marker genes was observed in the *mtmr8 *morphants.

Moreover, the effects of *mtmr8 *knockdown on vasculogenesis were judged by endogenous alkaline phosphatase activity. In Cont-MO embryos (Figure 4K, L), the endogenous alkaline phosphatase activity labeled the major cerebral vessels and the subintestinal vessels (SIVs). However, all Mtmr8 morphants displayed an obvious reduction in the head signal and a total absence of SIV labeling (Figure 4M, N). The obvious effects of *mtmr8 *knockdown on endothelial cell alkaline phosphatase activity further suggest that the vascular changes should be specific to endothelial cells, and the endothelial cell marker gene *fli1 *should be primary target for the *mtmr8 *functions.

### Mtmr8 mediates PI3K/Akt signaling for artery specification

**Figure 5:**
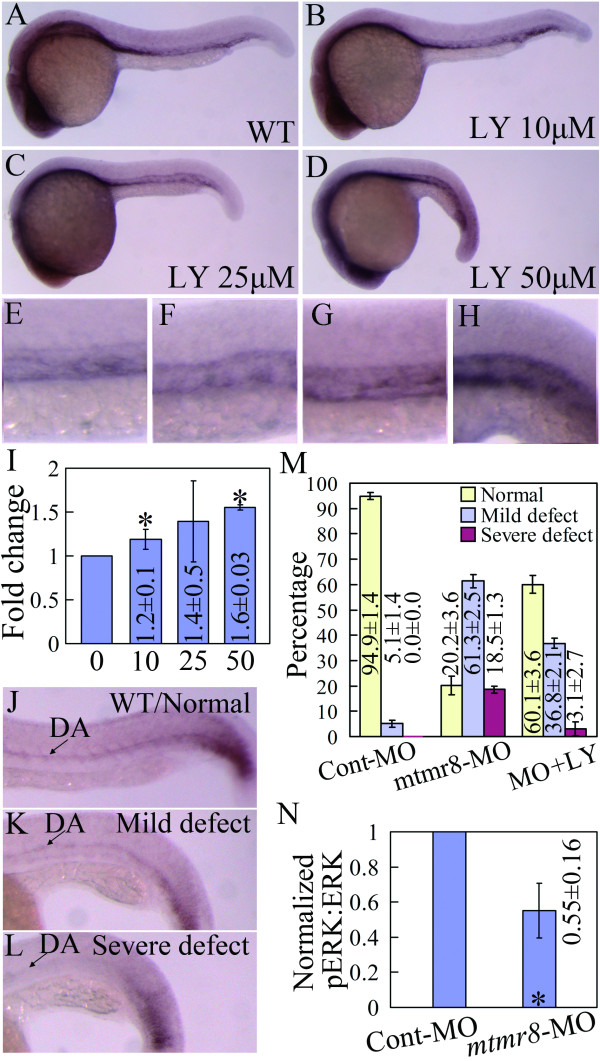
**Mtmr8 negatively regulates the PI3K/Akt pathway**. (A-D) *Mtmr8 *expression in wild type (WT) embryo (A) and the treated embryos (C-D) with different doses of LY294002 (LY) at 26 hpf. (E-H) The corresponding amplification of A-D, showing the detailed changes in the trunk vasculature. (I) *Mtmr8 *expression changes revealed by Q-RT-PCR in embryos treated with different doses of LY294002 (μM). (J-L) Three phenotypes of *ephrinB2a *expression and quantification data (M) in 26 hpf Mtmr8 morphants (30 embryos in each experiment group). (N) Quantitative Western blot data of ERK phosphorylation in 20-somite Mtmr8 morphants. * Indicates significance of *p *< 0.05.

In our previous study, *mtmr8 *knockdown was revealed to activate the PI3K/Akt pathway[19]. To determine whether the cooperation of Mtmr8 with PI3K plays a role during vasculogenesis, we firstly assayed the affect of PI3K inhibitor LY294002 on *mtmr8 *expression in zebrafish embryos. As shown in Figure 5, compared with control embryos (Figure 5A, E), lower concentration of LY294002 (10 and 25 μM) has no obvious effect on *mtmr8 *expression in wild-type embryos (Figure 5B, C, F, G), but significant increase expression of *mtmr8 *appears in the trunk vasculature when 50 μM LY294002 is used (Figure 5D, H). Figure 5I shows the quantitative data from Q-RT-PCR.

In *mtmr8 *morphants, the expression of artery marker gene *ephrinB2a *was severely reduced (Figure 4D) in the trunk region, but the reduced levels were variable, and might be classified into three kinds: normal, similar to consistent expression in wild-type (Figure 5J), mild defect, patchy expression in some cells along the DA (Figure 5K), severe defect, almost no any *ephrinB2a *expression in the trunk DA (Figure 5L). In previous studies[19], when treated with 10 μM LY294002, about half of the *mtmr8 *morphants could be further deteriorated in muscle development, and the others were mildly or not obviously affected (Data not shown). However, 4 μM LY294002 had no obvious effect on the muscle phenotype of the morphants. Interestingly, when 4 μM LY294002 was used to treat the *mtmr8 *morphants, a significantly increasing proportion of normal embryos was found in the morphants (Figure 5M). Subsequently, we checked phosphorylation status of another early arterial progenitor marker ERK (p42/44 MAP kinase)[18] in the *mtmr8 *morphants by Western blot analysis, and found that loss of Mtmr8 function significantly reduces ERK phosphorylation (Figure 5N). The data indicate that Mtmr8 negatively mediates PI3K/Akt signaling for artery specification.

### Mtmr8 is essential for normal vasculature development through regulating Hedgehog and Vegf signaling pathways

**Figure 6:**
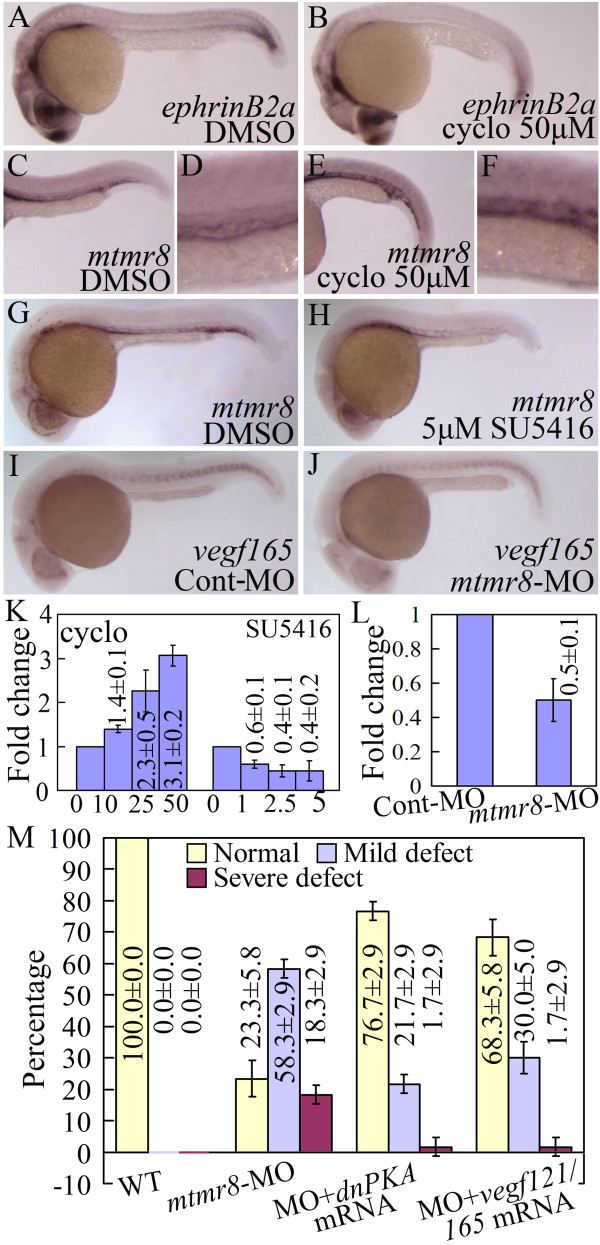
**(A-F) The expression of *ephrinB2a *(A, B) and *mtmr8 *(C-F) in 26 hpf embryos incubated with DMSO (A, C) or cyclopamine (B, E)**. (D, F) are magnification of (C, E) in the trunk vasculture. (G-H) *Mtmr8 *expression in 30 hpf embryos incubated with DMSO (G) or SU5416 (H). (I-J) *Vegf165 *expression in 26 hpf Cont-MO and *Mtmr8 *morphant embryos. (K) *Mtmr8 *expression changes revealed by Q-RT-PCR in embryos treated with different doses of SU5416 and cyclopamine (μM). (L) *Vegf165 *expression in 26 hpf Cont-MO and Mtmr8 morphant embryos revealed by Q-RT-PCR. (M) Statistical data of different defect phenotypes at 26 hpf in Mtmr8-MO, Mtmr8-MO+dnPKA mRNA, and Mtmr8-MO+vegf121/165 mRNA embryos.

Hh signaling is multi-functional, and its role is temporal-spatially determined during embryogenesis[26]. Shh from the notochord promotes Vegf expression by the adjacent somite, which promotes expression of the artery-specific EphrinB2 in the dorsal aorta[27]. To determine the position of Mtmr8 in this regulatory cascade, we tested its expression in embryos when these pathways were inhibited. As shown in Figure 6A and 6B, in 26 hpf wild-type embryos, *ephrinB2a *is absent in the dorsal aorta when exposed to 50 μM cyclopamine, and *vegf *expression is lost within the somites but persisted within the hypochord (Data not shown, the result same to Lawson et al[9]), which means that inhibition of Hedgehog signaling can partially disrupt Vegf expression. Then, we investigated whether cyclopamine could also inhibit the *mtmr8 *expression. As shown in Figure 6C-F, *mtmr8 *shows stronger expression in the posterior cardinal vein than in the dorsal aorta of wild-type control embryos (Figure 6C-D), and its expression was obviously upregulated in the cyclopamine-treated embryos (Figure 6E-F). Figure 6K shows the gradient up-regulation of *mtmr8 *under different doses of cyclopamine obtained from Q-RT-PCR.

Vascular function is tightly regulated by Vegf signaling pathway[28]. To assess whether Mtmr8 influenced the developing vasculature through Vegf pathway, we treated 10 hpf embryos with a Vegf receptor tyrosine kinase inhibitor, SU5416. In comparison with control embryos (Figure 6G), the embryos incubated with 5 μM of SU5416 failed to show vascular expression of *mtmr8 *in the trunk at 30 hpf (Figure 6H), and the reduced transcript levels were detected under different doses of SU5416 by Q-RT-PCR (Figure 6K), suggesting that Mtmr8 might act downstream of Vegf to determine arterial cell fate. Moreover, we checked the expression of *vegf165 *in *Mtmr8 *morphants and control embryos, and found a significant down-regulation of *vegf165 *expression within the somites at 26 hpf (Figure 6I, J), and the quantitative down-regulation change was confirmed by Q-RT-PCR (Figure 6L).

**Figure 7:**
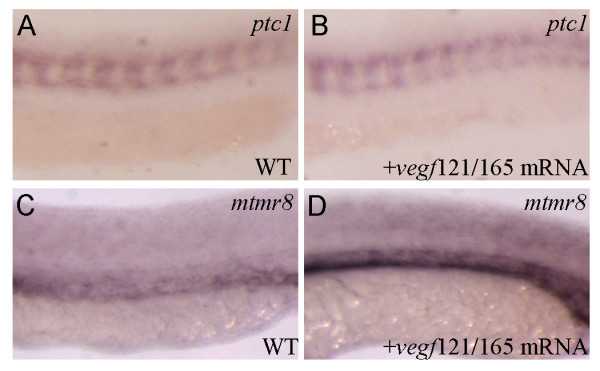
**The effect of Vegf overexpression on *mtmr8 *expression**. (A, B) Expression of *ptc1 *in wild type (WT) embryo (A) and in vegf121/165 mRNA injected embryo (B). (C, D) Expression of *mtmr8 *in wild type (WT) embryo (C) and in vegf121/165 mRNA injected embryo (D). All embryos in 25 hpf; anterior toward the left.

In zebrafish, over-expression of either *vegf121 *or *vegf165 *could rescue arterial differentiation blocked by a deficiency of Shh signaling[9]. Direct activation of PKA, an inhibitor of Hedgehog signaling, induces endothelial cell apoptosis and inhibits angiogenesis *in vivo*, and suppressing the PKA activity by expression of dnPKA, promotes endothelial cell survival and migration during angiogenesis[29,30]. Mtmr8 acts downstream of Vegf to determine arterial cell fate, and its knockdown can impair the Hedgehog signaling. On this basis, we further assessed whether exogenous Vegf and Hedgehog signaling were sufficient to rescue *ephrinB2a *expression in the absence of *mtmr8 *activity. As shown in Figure 6M, when injected with 100 pg *dnPKA *or *vegf121*+*vegf 165 *mRNA, the *ephrinB2a *expression in *mtmr8 *morphants can be rescued as shown by reducing percentage of embryos in the severe or mild defect group, which can not be rescued by control GFP mRNA (Data not shown). Moreover, we tested whether Vegf overexpression would lead to increase of *mtmr8 *expression. As shown in Figure 7, when 100 pg *vegf121*+*vegf 165 *mRNA was injected, *ptc1 *expression is not affected (Figure 7A, B), but the expression of *mtmr8 *was up-regulated (Figure 7C, D) (n = 17/28) in the injected embryos. We therefore conclude that Mtmr8 expression in vasculature is dependent on Vegf, and Mtmr8 is essential for normal vasculature development through regulating Hedgehog and Vegf signaling pathways.

## Discussion

Previous studies have showed that mutations in most myotubularin family genes are causative for human neuromuscular disorders[31]. And, knockdown of zebrafish *myotubularin *and *mtmr8 *also impairs the embryo muscle development[19,32]. Moreover, Mtmr8 has been shown to negatively regulate the PI3K/AKT pathway[19]. In the current study, we have further found that *mtmr8 *knockdown leads to activation of PI3K/Akt signaling, which impairs arterial endothelial marker gene expression, and results in endothelial cell impairment and vasculogenesis defects. Significantly, *mtmr8 *expression has been detected in somitic and lateral mesoderm, where the angioblasts are committed to endothelial lineage differentiation and restricted to arterial or venous fate[19,33]. And, significant expression reduction of endothelial progenitor marker genes in *mtmr8 *morphants indicates that the impaired arterial differentiation should be the consequence of defects in endothelial cell formation. These results suggest that *mtmr8 *should be essential for the endothelial cell differentiation and vasculature development through repressing the activity of PI3K in zebrafish.

**Figure 8:**
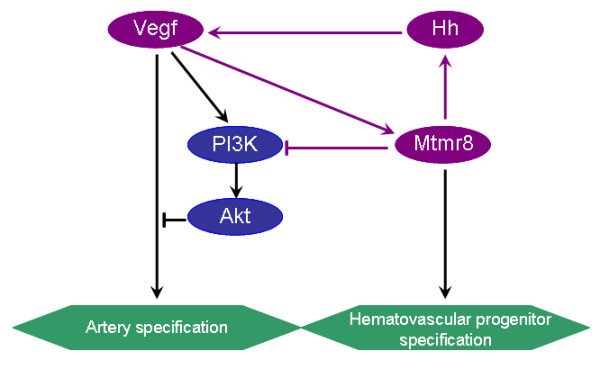
**A hypothesized signal pathway that Mtmr8 regulates artery specification and hematovascular progenitor specification in zebrafish embryo**. Mtmr8 inhibits PI3K/Akt pathway, and activates Hh and Vegf signaling pathway, which may interact with each other in regulating vasculature development. The pink arrows indicate the regulatory loop.

Hedgehog signaling from notochord has been demonstrated to promote somitic expression of Vegf, and thereby to promote expression of the artery-specific EphrinB2 in dorsal aorta[27]. *Mtmr8 *expression has been detected in somitic and lateral mesoderm, where angioblasts are restricted to arterial or venous fate, suggesting that the impaired arterial differentiation in *mtmr8 *morphants might be the consequence of defects in Hedgehog/Vegf signaling pathways. And, the expression of *mtmr8 *is significantly repressed in the embryos treated with inhibitor of Vegf signaling pathway (Figure 5G-H), suggesting that Mtmr8 might act downstream of Vegf to determine arterial cell fate. The concomitant down-regulation of *ephrinB2a*, *ptc1 *and *vegf *in Mtmr8 morphants raises the possibility that Mtmr8 acts upstream of Hedgehog pathway. We hypothesize that Mtmr8 regulates artery differentiation through two parallel ways (Figure 8). In one way, Mtmr8 may act downstream of Vegf. In other way, Mtmr8, Hedgehog and Vegf may control artery specification through a regulatory loop (Figure 8). In normal physiological condition, Mtmr8 is a downstream factor of Vegf signaling pathway, and is partially regulated by the Hedgehog signaling pathway that is affected by the expression of Mtmr8. Additionally, the down-expression of the hematovascular progenitor marker genes *hbae1 *and *fli1 *in *mtmr8 *morphants (Figure 1) suggests that *mtmr8 *might also regulate hematovascular progenitor specification and differentiation (Figure 8).

## Conclusion

In conclusion, our findings indicate that Mtmr8 functions as a key regulator of somitic muscle development and vasculature development in zebrafish embryos. The signaling pathways that function during vascular development are highly conserved. Further analysis for Mtmr8 function will be essential to understand the complex signaling hierarchy for the differentiation of endothelial cells and the development of vasculature in vertebrates.

## Methods

### Zebrafish husbandry

*Danio rerio *wild-type (WT) AB strain, and *Tg(kdrl:GFP)^la116 ^*transgenic zebrafish (previous *Tg(flk1:GFP)^la116^*) [23] were used. Zebrafish were maintained and staged as described[34]. When necessary, embryos were anesthetized with 0.003% tricaine (Sigma). All of the experimental researches on zebrafish were performed with the approval of the animal ethics committee in the Institute of Hydrobiology, Chinese Academy of Sciences.

### Antisense morpholino and mRNA microinjection

The sequences and doses of the injected morpholinos were described previously[19]. *mtmr8*, *dnPKA, scl, vegf121 *and *vegf165 *cDNAs were subcloned into PCS2^+ ^plasmid for *in vitro *synthesis of capped mRNA, using SP6 RNA polymerase (Ambion), following the manufacturer's instructions. A mis-matched *mtmr8 *mRNA (mis-*mtmr8*) was constructed by using mis-match forward prime 5'-ATGGAGCACATCATAACGCCCAAAGTCG-3' (underlines indicate the changed nucleotide). All mRNA were re-suspended in water and co-injected at a concentration of 50-100 ng/μL.

### Whole-mount in situ hybridization, endogenous alkaline phosphatase-based vessel staining and histological analysis

Embryos at different stages were collected and fixed as described[35]. Purified plasmids were linearized by selected restriction enzymes and used as templates for *in vitro *transcription using T7 or Sp6 RNA polymerase to generate DIG-labeled (Roche) sense and anti-sense probes. Whole mount *in situ *hybridization was performed as described previously[36,37].

At 72 hpf, embryos were fixed in 4% paraformaldehyde and dehydrated in methanol. Later, they were rehydrated to PBST and then used for staining, as described by Serbedzija *et al *[38]. Histological analysis was performed according to methods described by Lawson *et al *[17].

### Real-time quantitative PCR (Q-RT-PCR) and Western blot detection

Real-time quantitative PCR was performed by DNA Engine Chromo 4 Real-Time System (MJ Research) with SYBR Green I Dye according to a previous report[39]. The ratio of these genes to *β-actin *in control embryos was set to 1 (100%), and the expression of all morpholino-injected or drug-treated embryos were normalized relative to this value. All samples were analyzed in triplicates and the results were expressed relative to the expression of *β-actin *using the 2 (-delta delta C(T)) method [40]. Data are presented as mean ± SD of three separate experiments.

Western blot detection for the ratio between phospho-p44/42 MAP kinase (Thr202/Tyr204) and p44/42 MAP kinase in developing zebrafish larvae was performed essentially as described[18]. The membranes were washed and incubated with Alkaline Phosphatase (AP)-conjugated secondary antibodies. Images of blots were captured with a scanner, and quantitative densitometric analysis was performed using Scion Image.

### Drug treatments

PI3K inhibitor, LY294002 (Promega) was dissolved in DMSO at stock concentration of 50 mM. For experiments, embryos were incubated in embryo media containing 0-50 μM of the drug from 10 hpf to 24 hpf. Control embryos were treated with the equivalent amount of DMSO solution (1%≤).

Cyclopamine and SU5416 were obtained from Calbiochem and were used at a concentration of 0-50 μm and 0-5 μM respectively. Zebrafish embryos were soaked in 6-well plates and were treated with the drugs from 10 hpf, until they were processed for *in situ *experiments. Control embryos were treated in embryo medium containing equivalent amount of DMSO solution (1% < ).

### Statistical analysis

All the experiments were performed in triplicates (about 30 embryos in each experiment group). Data were presented as mean ± SD. The data in morphant embryos compared to the control (set to 1) were assessed by one-way analysis of variance (ANOVA), followed by the Tukey's post hoc test for multiple comparisons. A probability (*P) of <0.05 was considered statistically significant.

## Authors' contributions

Contribution: JF Gui and J Mei designed research; J Mei, S Liu and Z Li performed research; JF Gui, J Mei and S Liu analyzed the data and wrote the paper. All authors read and approved of the final manuscript.

## Supplementary Material

Additional file 1 **The effects of *mtmr8 *knockdown on circulation of embryos at 28 hpf**. Movie showing circulation in trunk of a Cont-MO injected embryo (left side) and loss of circulation in trunk of a *mtmr8 *morphant embryo at the same stage (right side). Anterior is to the up. Click here for file

Additional file 2 **The effects of *mtmr8 *knockdown on circulation of embryos at 36 hpf**. Movie showing circulation in trunk of a Cont-MO injected embryo (left side) and loss of circulation in trunk of a *mtmr8 *morphant embryo at the same stage (right side). Anterior is to the up. Click here for file

Additional file 3 **The effects of *mtmr8 *knockdown on heart contractions of embryos at 48 hpf**. Movie showing heart contractions of a Cont-MO injected embryo (left side) and a *mtmr8 *morphant embryo at the same stage (right side). Click here for file
